# Some novel antileishmanial compounds inhibit normal cell cycle progression of *Leishmania donovani* promastigotes and exhibit*s* pro-oxidative potential

**DOI:** 10.1371/journal.pone.0258996

**Published:** 2021-11-22

**Authors:** Wandayi Emmanuel Amlabu, Cynthia Mmalebna Amisigo, Christine Achiaa Antwi, Gordon Akanzuwine Awandare, Theresa Manful Gwira

**Affiliations:** 1 West African Centre for Cell Biology of Infectious Pathogens, University of Ghana, Accra, Ghana; 2 Department of Zoology, Faculty of Life Sciences, Ahmadu Bello University, Zaria, Nigeria; 3 Department of Biochemistry, Cell and Molecular Biology, University of Ghana, Accra, Ghana; National Centre For Cell Science, INDIA

## Abstract

In the midst of numerous setbacks that beclouds the fight against leishmaniasis; a neglected tropical disease, the search for new chemotherapeutics against this disease is of utmost importance. Leishmaniasis is a disease closely associated with poverty and endemic in Africa, Asia, southern Europe and the Americas. It is caused by parasites of the genus *Leishmania* and transmitted by a sandfly vector. In this study, we evaluated the antileishmanial potency of eighteen pathogen box compounds and elucidated their biosafety and possible mechanisms of action against *Leishmania donovani* promastigotes and amastigotes *in vitro*. IC_50_s range of 0.12±0.15 to >6.25 μg/ml and 0.13±0.004 to >6.25μg/ml were observed for the promastigotes and amastigotes, respectively. We demonstrated the ability of some of the compounds to cause cytocidal effect on the parasites, induce increased production of reactive oxygen species (ROS), disrupt the normal parasite morphology and cause the accumulation of parasites at the DNA synthesis phase of the cell cycle. We recommend a further *in vivo* study on these compounds to validate the findings.

## Introduction

*Leishmania* sp. were independently described by William Leishman and Charles Donovan in 1903 and the genus proposed by James Wright in 1903 [1, 2]. They belong to the Class: Kinetoplastida and Family: Trypanosomatidae. These parasites have a dimorphic life cycle with the organism shuttling between a flagellated free-living promastigote in the gut of the sand fly vector and an intracellular amastigote in the mammalian host [3, 4].

Principally, *Leishmania* species are parasites of vertebrate organisms with some serving as definitive hosts (e.g in humans) and/or reservoir hosts (e.g cats, dogs, rodents etc) which are needed for maintaining the transmission cycle even when disease morbidity are low amongst the human populations. In humans, *Leishmania* causes a spectrum of diseases referred to as leishmaniasis. This disease is presented in four different forms with clinical conditions that ranges from the cutaneous leishmaniasis (CL- ulcerative localized skin lesions), to the diffuse cutaneous leishmaniasis (DCL- multiple non ulcerative nodules), the mucocutaneous leishmaniasis (ML-a destructive mucosal inflammation) and finally, the visceral leishmaniasis (VL-which affects the visceral organs). This wide spectrum of clinical manifestations of the disease makes its diagnosis often difficult as it shares some similar etiological presentation with diseases like leprosy, skin cancers for CL and malaria / schistosomiasis for VL [5].

Leishmaniasis, being one of the neglected tropical diseases (NTDs), is a disease associated with poverty and endemic to the tropical and subtropical regions of the world particularly in Africa, Asia, South America and southern Europe [6]. About 12 million humans are infected over 350 million people are at risk of infection and 700,000–1.5 million new cases are reported annually [7, 8]. There are about 70,000 deaths due to this disease annually, although this is probably an underestimate because leishmaniasis is a reportable disease in only 40 of the 88 endemic countries and many *Leishmania* infections can be asymptomatic or misdiagnosed [9].

Interventions are largely through chemotherapy owing to the fact that no vaccines are yet available against leishmaniasis. Some of the main drugs that are used for leishmaniasis are pentamidine, pentavalent antimonials, miltefosine, paromomycin and amphotericin B. However, they are laden with numerous setbacks which include, adverse side effects, high cost, non-availability, lengthy treatment time and high toxicity [10–12].

The Pathogen Box contains 400 diverse, drug-like molecules active against neglected diseases of interest and were assembled by Medicine for Malaria Venture (MMV) with the aim of accelerating drug development for poverty-related diseases. In this study, 18 of the compounds were selected because they have been found to be ‘kinetoplastid sensitive’ as designated by MMV. These compounds were tested for their antileishmanial potency against *Leishmania donovani* and their possible mode of action explored in regard to the morphological changes they induce, their apoptotic and necrotic potentials, the level of reactive oxygen species generation and their effect on the normal cell cycle progression of the promastigote form of the parasite in a bid to elucidate their promise as new leads for chemotherapeutic interventions against leishmaniasis.

## Materials and methods

### Pathogen box compounds

The eighteen (18) pathogen box compounds screened in this study are: MMV688793 (D05A), MMV688514 (E02A), MMV687762 (D02A), MMV688943 (C06A), MMV688796 (A06A), MMV690028 (C05A), MMV690027 (C02B), MMV676600 (C03B), MMV676602 (H02B), MMV676162 (C06D), MMV676159 (A07E), MMV688797 (E05A), MMV688958 (G05A), MMV688360 (H03A), MMV099637 (H04A), MMV688798 (H05A), MMV652003 (A03B) and MMV676604 (B03B). A Stock solution of 10 mM in 100% DMSO of each compound was obtained from the pathogen box and a working solution was formulated with double distilled water (ddH_2_O). The final DMSO concentration for each working solution was less than 0.4%. Amphotericin B (Sigma Aldrich, USA), an established antileishmanial was used as a positive control and was reconstituted in double distilled water (ddH_2_O).

### *In vitro* cultivation of *Leishmania donovani* and RAW 264.7 cell line

Promastigotes of *Leishmania donovani* and the murine macrophage cell line RAW 264.7 were cultured in M199 media and DMEM respectively and maintained under the same conditions as described by [13].

### Antileishmanial activity and determination of inhibitory concentrations for promastigotes and macrophage-amastigotes

*Leishmania donovani* promastigotes parasites at a density of 2 x 10^5^ cells/ml were incubated without or with the compounds in triplicates, at varying concentrations (0.0156- >6.25 μg/ml) concentrations and incubated at 25° C for 96 hrs to determine the IC_50_, i.e., the concentration of the compound that inhibits growth of 50% population of the parasites. Amphotericin B was used as a positive control. The 3-(4,5-Dimethylthiazol-2-yl)-2,5-diphenyltetrazolium bromide (MTT) assay was used to determine the parasite viability as previously described [13].

The RAW macrophage cells were infected with promastigotes at a density of 1:10 ratio, respectively for 12 hrs and the non-phagocytosed promastigotes and media removed by aspiration. The RAW macrophages cells containing the internalized amastigote cells were treated with the compounds to test for their growth inhibitory potentials as previously described [14]. The 3-(4,5-Dimethylthiazol-2-yl)-2,5-diphenyltetrazolium bromide (MTT) assay was also used to determine the parasite viability. Fluorescence was measured with a Varioskan Lux Elisa plate reader at a wavelength of 570 nm. The 50% inhibitory concentrations (IC_50s_) were determined as previously described [15–17].

### Cytotoxicity of the compounds against the mammalian RAW macrophage cell line

RAW 264.7 a murine macrophage cell line was cultured in DMEM supplemented with penicillin G sodium and streptomycin sulfate both at a concentration of 100 μg/ml, and 10% FBS. The mammalian cells at a density of 1 x 10^6^ cells/ml were incubated at different concentrations of the compounds ranging from 0.39–100 μg/ml for 48 hrs. Here, amphotericin B was used as a positive control and the negative control was untreated RAW cell line. Using the MTT assay, the 50% cytotoxic concentration (TC_50_) of the compounds were determined by analysis of dose-response curves as was done previously [13, 14], and the therapeutic or selectivity index was calculated [15].

### Haemolytic potentials of the compounds

The compounds were tested against the O^+^ human red blood cells (obtained from the Malaria laboratory of WACCBIP) at a density of 2% haematocrit for their ability to lyse erythrocytes upon incubation [18]. Here, a concentration of up to 100 μg/ml of compounds was used, and the 50% haemolytic concentration (HC_50_) of the compounds were determined by analysis of dose-response curves as described [14].

### Growth kinetics and cytocidal/cytostatic activity

The compounds (at 1 μg/ml) were tested for their effects on the growth kinetic pattern against the promastigotes of *L*. *donovani*. The parasite cells (2 x 10^5^ cells/ml) were incubated at 25° C in the presence of the test compounds in M199 supplemented with 10% FBS. Amphotericin B treated parasites (0–50 μg/ml) were used as the positive control while the untreated promastigotes in medium alone served as the negative control. Viable cells were counted every 24 hrs for a total of 120 hrs as indicated in previous studies [14, 16].

The cytocidal/cytostatic effect of the test compounds were assessed by analyzing the treated and untreated parasites after 120 hrs of incubation. Cells were washed twice with fresh M199, resuspended in complete M199 media and incubated further for 96 hrs. The viable cells were then counted microscopically [14, 16, 19].

### Measurement of ROS levels in *Leishmania donovani* treated with MMV compounds

The degree of reactive oxygen species (ROS) production in treated promastigote and amastigote cells of *L*. *donovani* were assessed using the H_2_DCFDA (2’,7’-dichlorodihydrofluorescein diacetate) rich fluorescent dye (MAK145 Sigma-Aldrich) ROS detection kit. Cells at a density of 2 x 10^5^ cells/ml were treated with the MMV compounds at a concentration of 100, 50 or 25 μM in triplicates for 72 hrs (in the case of promastigotes) and 24 hrs (in the case of amastigotes). Amoxicillin was used as a positive control for pro-oxidative generation while Vitamin C was used as a positive control for anti-oxidative potential. The treated and untreated cells were then suspended in PBS, washed, and resuspended in PBS, then incubated with the ROS detection master mix for 1 hr as instructed by the protocol. The cells were analyzed for intracellular ROS generation by measuring the fluorescence intensity which is proportional to the ROS production level using the Varioskan Lux Elisa microplate reader (Thermo Fischer Scientific, USA) at a wavelength of 605 nm [20, 21].

### Externalization of phosphatidylserine (PS) in the outer membranes of *Leishmania donovani* promastigotes treated with the MMV compounds

A double staining of treated/ untreated promastigotes of *Leishmania donovani* (2 x 10^5^ cells/ml) with Annexin V-FITC and Propidium Iodide (PI) was done to determine the apoptotic and necrotic effects of the some of the established cytocidal compounds amongst the eighteen MMV compounds studied. Treatment was set at 2x IC_50_ concentrations of the respective test compounds against a negative control of untreated promastigote cells in a 24 hrs incubation period. The cells were then prepared using the Annexin V-FITC apoptosis detection kit following the Sigma protocol (Catalog Number: APOAF). Further treatment and analysis of cells was done as shown previously [14].

### Cell cycle analysis

The analysis was done using the protocol as designed in the Guava cell cycle reagent manual (catalog No. 4500–0220). Compounds were tested at 2x IC_50_ concentrations on promastigotes (density of 2 x 10^5^ cells/ml). Both treated and untreated *Leishmania* cells were incubated for 24 hrs, samples read using a flow cytometer (BD LSR Fortessa X-20 analyzer BD Biosciences), and the respective cell counts analyzed in the G0/G1, S and G2/M Phases. Data generated was further analyzed using the FlowJo V10 software [14].

### Analysis of morphological and mitochondrion effects of compounds by fluorescence microscopy

Both untreated and treated promastigotes at a cell density of 2 x 10^5^ cells/ml (2x IC_50_ drug concentration) were incubated for 24 hrs and 72 hrs to study the morphological changes due to the effect of the test compounds. Their mitochondrial integrity were analyzed using a slightly modified DAPI and Mitotracker dye protocol as described by Amisigo and colleagues [22].

### Statistical analysis

The IC_50_, TC_50_ and HC_50_ were obtained from dose-response curves using Microsoft Excel. All data are expressed as mean ±standard deviation (SD) of three independent experiments. ANOVA was used to measure the statistical significance (P<0.05) between treatment groups in the growth kinetic and reversibility assays using the Graph Pad Prism 6.0 Software. Effects of compounds on cell cycle and apoptosis were obtained by flow cytometry and the data analyzed using FlowJo V10 software. Student t-test was used to determine their statistical significance at P<0.05.

## Results

### Growth inhibitory profile and selectivity index of promastigotes and macrophage-amastigotes models of *Leishmania donovani*

The growth inhibitory potential and selectivity of the following MMV compounds against the promastigotes (SI 1 and SI 2) and amastigotes (SI 3 and SI 4) of *Leishmania donovani* were evaluated *in vitro*; MMV688793 (D05A), MMV688514 (E02A), MMV687762 (D02A), MMV688943 (C06A), MMV688796 (A06A), MMV690028 (C05A), MMV690027 (C02B), MMV676600 (C03B), MMV676602 (H02B), MMV676162 (C06D), MMV676159 (A07E), MMV688797 (E05A), MMV688958 (G05A), MMV688360 (H03A), MMV099637 (H04A), MMV688798 (H05A), MMV652003 (A03B) and MMV676604 (B03B). These are among the 68 kinetoplastid designated compounds found in the MMV pathogen box. The most active compounds against the promastigotes were 12 of the 18 compounds with IC_50_s ranging between 0.12–0.39 μg/ml, while the least active were 6 with IC_50_s ranging between 0.44 - >6.25 μg/ml (Table 1). Four (4) of the compounds were active against both forms of the parasite. The selectivity of the compounds against the parasites were evaluated by testing their cytotoxicity against the murine macrophage cell lines RAW (SI 5 and SI 6) and a therapeutic index range of 0.062–666 and 0.017–454 was obtained for promastigotes and amastigotes respectively; showing the biosafety of some of the compounds against non-target cells *in vitro* (Table 1). Also, the haemolytic profile of the compounds (SI 7 and SI 8) indicated their non- haemolytic effect except one (C05A MMV690028) against the human red blood cell corpuscles (Table 1).

**Table 1:** The 50% inhibitory concentration (IC_50_) of the compounds, their selectivity against the promastigotes/amastigotes of *Leishmania donovani* and their haemolytic profile (HC_50_).

S/NO	Compound code	IC_50_ (μg/ml) ±SD promastigote	TC_50_ (μg/ml) ±SD RAW	Therapeutic Index (TC_50_/IC_50_) promastigotes	IC_50_ (μg/ml) ±SD amastigote	Therapeutic Index (TC_50_/IC_50_) amastigotes	HC_50_ (μg/ml)
1	A06A	0.19±0.008	0.39±0.05	2.05	0.38±0.010	1.03	>100
2	C02B	0.15±0.130	0.38±0.010	2.53	0.13±0.004	2.92	>100
3	C03B	0.62±0.010	0.21±0.010	0.34	>6.25	0.03	>100
4	H02B	0.12±0.150	0.11±0.010	0.92	>6.25	0.02	>100
5	C05A	0.95±0.020	>100	105.26	>6.25±0.030	16	5.5
6	C06A	0.21±0.008	>100	476.19	4.6±0.004	21.74	>100
7	D02A	0.21±0.010	>100	476.19	0.22±0.020	454.55	>100
8	D05A	0.20±0.020	>100	500	3.8±0.030	26.32	>100
9	E02A	0.29±0.001	>100	344.83	5.34±0.010	18.73	>100
10	C06D	>6.25	0.39±0.010	0.06	3.0±0.001	0.13	>100
11	A07E	>6.25	0.41±0.020	0.07	>6.25	0.07	>100
12	E05A	0.39±0.060	>100	256.41	2.74±0.100	36.50	>100
13	G05A	0.15±0.020	>100	666.67	2.1±0.010	47.62	>100
14	H03A	0.15±0.030	>100	666.67	2.69±0.100	37.17	>100
15	H04A	0.31±0.008	>100	322.58	>6.25	16	>100
16	H05A	0.78±0.170	0.15±0.030	0.19	0.25±0.040	0.60	>100
17	A03B	0.44±0.200	0.18±0.050	0.41	0.20±0.030	0.90	>100
18	B03B	0.20±0.040	0.25±0.040	1.25	0.40±0.050	0.63	>100

TC_50_ = Toxic concentration against 50% of cell population, HC_50_ = Haemolytic concentration against 50% of cell population. All data represent the mean of three independent experiments.

### The cytocidal/cytostatic effects and the growth kinetic patterns of the test compounds against the promastigotes of *Leishmania donovani*

**Fig 1:**
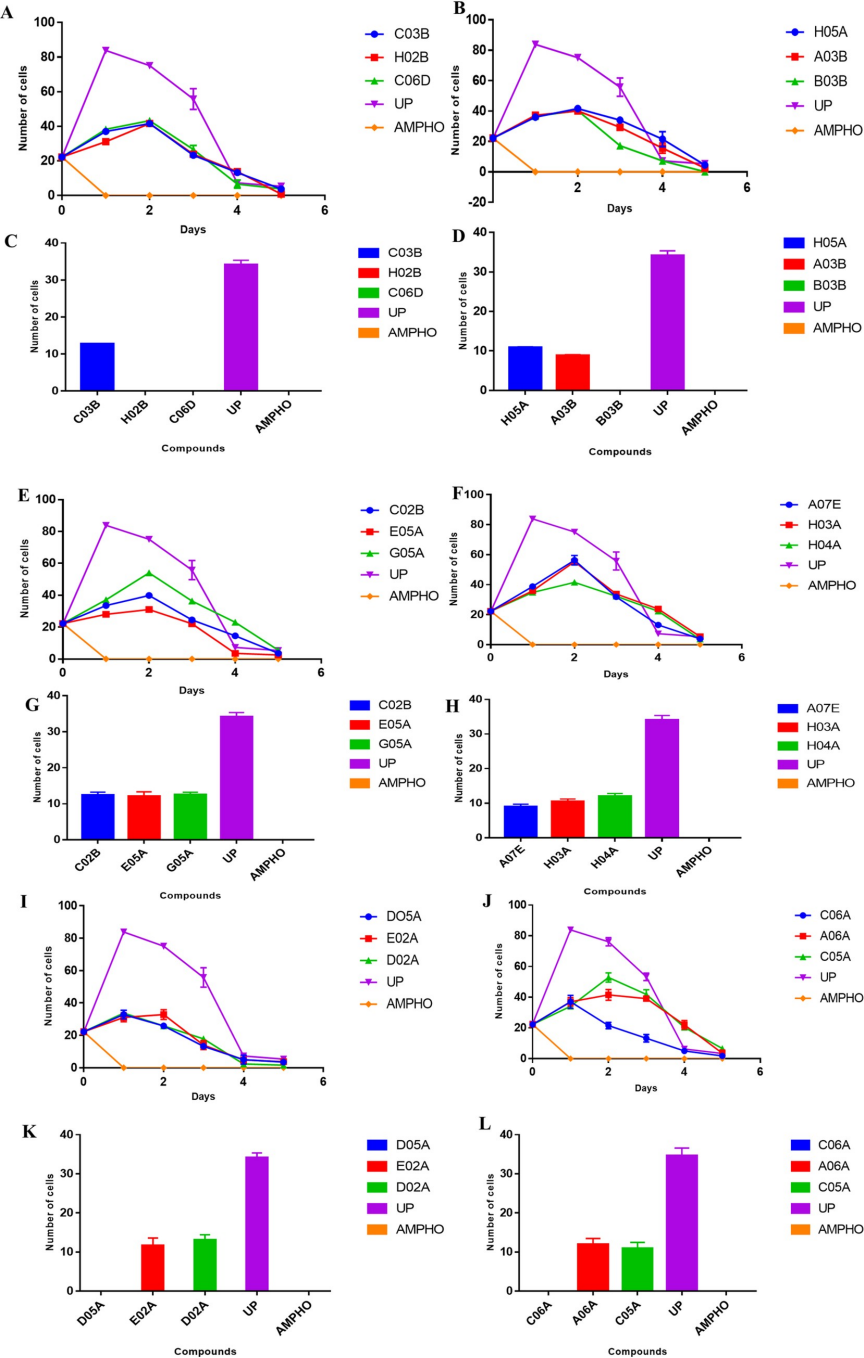
Growth kinetics, cytocidal and cytostatic profile of the MMV compounds on *Leishmania donovani* parasites. The growth kinetics curves of (**A**) MMV676600 (C03B), MMV676602 (H02B) and MMV676162 (C06D), (**B**) MMV688798 (H05A), MMV652003 (A03B) and MMV676604 (B03B), (**E**) MMV690027 (C02B), MMV688797 (E05A) and MMV688958 (G05A), (**F**) MMV676159 (A07E), MMV688360 (H03A) and MMV099637 (H04A), (**I**) MMV688793 (D05A), MMV688514 (E02A) and MMV687762 (D02A), (**J**) MMV688943 (C06A), MMV688796 (A06A) and MMV690028 (C05A) against *Leishmania donovani* promastigotes cells counted within 5 days of incubation. A concentration of 1μg/ml of the MMV compounds were used. ANOVA was used to analyze the significant differences between treatments at P<0.05. The growth reversibility profile of (**C**) MMV676600 (C03B), MMV676602 (H02B) and MMV676162 (C06D), (**D**) MMV688798 (H05A), MMV652003 (A03B) and MMV676604 (B03B), (**G**) MMV690027 (C02B), MMV688797 (E05A) and MMV688958 (G05A), (**H**) MMV676159 (A07E), MMV688360 (H03A) and MMV099637 (H04A), (**K**) MMV688793 (D05A), MMV688514 (E02A) and MMV687762 (D02A), (**L**) MMV688943 (C06A), MMV688796 (A06A) and MMV690028 (C05A) after drug withdrawal and resuspension of the cells in fresh complete media and cells counted after four days incubation. Data shows the mean of three independent experiments. UP = Untreated Parasites (Negative control) and AMPHO = amphotericin B (positive control).

The growth inhibitory pattern of the parasites for the eighteen selected compounds were elucidated and it showed that there were inhibitory effects on the promastigotes of *Leishmania donovani* within the span of 5 days compared with that of the untreated parasites (negative control) (Fig 1A and 1B). Upon drug withdrawal (growth reversibility assay), five of the compounds [MMV676604 (B03B), MMV676602 (H02B), MMV676162 (C06D), MMV688793 (D05A) and MMV688943 (C06A)] showed a cytocidal effect on the parasites while the remaining thirteen showed a cytostatic effect on the parasites with a growth reversal percentage ranging from 23–34% (Fig 1C and 1D).

### Effect of the treatment of *Leishmania donovani* with MMV compounds on ROS generation

**Fig 2:**
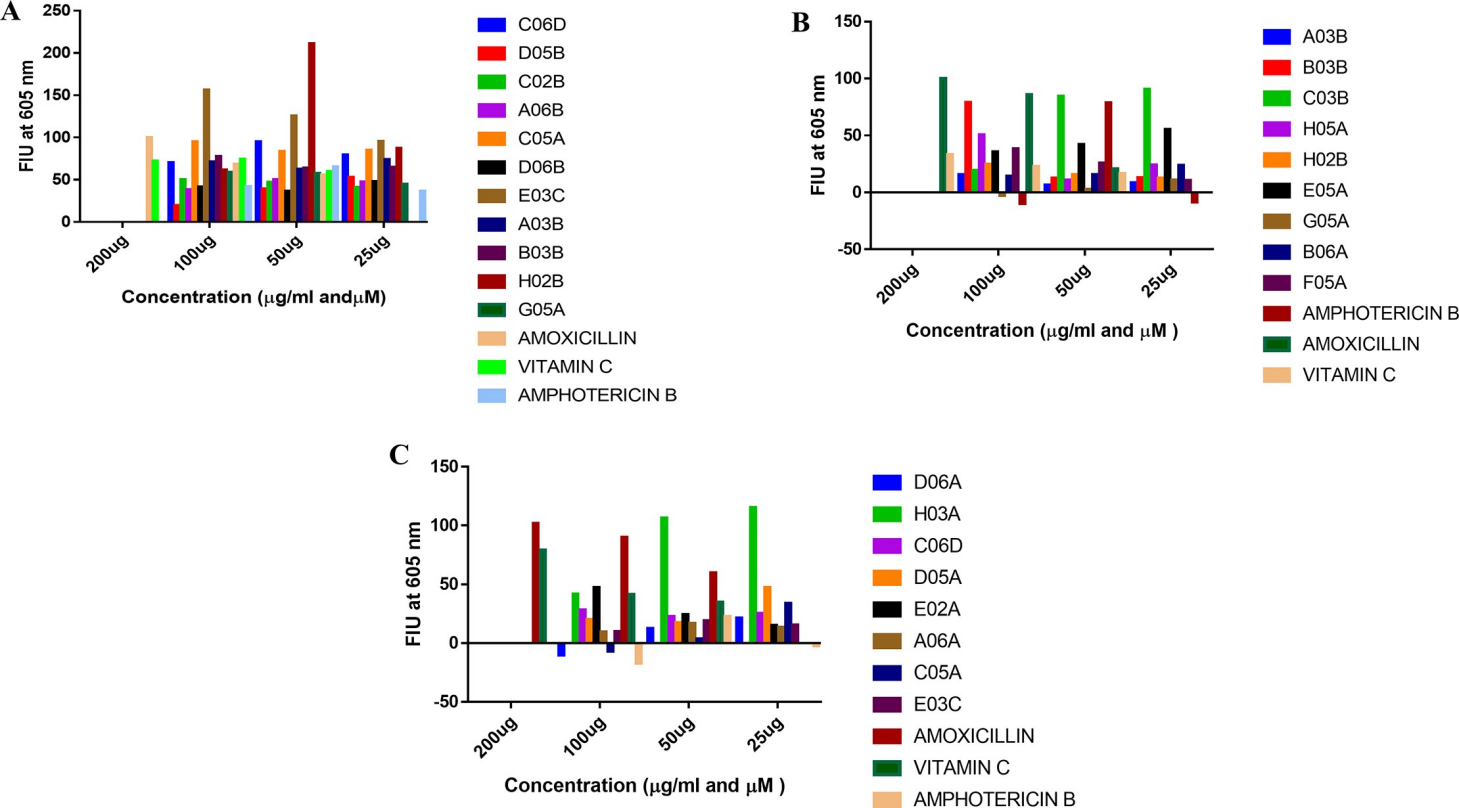
The effects of MMV compounds on ROS generation in amastigotes and promastigote of *Leishmania donovani*. **(A)** Pro-oxidative and anti-oxidative profiles of MMV compound treated amastigotes shown as an index of the fluorescence intensity unit of the H_2_DCFDA rich dye **(B and C)** Pro-oxidative and anti-oxidative profile of MMV compound treated promastigotes shown as an index of the fluorescence intensity unit of the H_2_DCFDA rich dye.

Reactive oxygen species (ROS) production in treated promastigote and amastigote cells of *L*. *donovani* were assessed using the H_2_DCFDA (2’,7’-dichlorodihydrofluorescein diacetate) rich fluorescent dye (MAK145 Sigma-Aldrich). Amoxicillin and Vitamin C were used as pro-oxidative and anti-oxidative positive controls, respectively. Treatment of amastigotes with Vitamin C, Amphotericin B, MMV688958 (G05A), MMV689255 (D06B), MMV688854 (A06B), MMV690027 (C02B), MMV000063 (D05B), MMV652003 (A03B) and MMV676604 (B03B) showed an anti-oxidative effect (Fig 2A) while Amoxicillin, MMV676602 (H02B), MMV676057 (E03C), MMV690028 (C05A), and MMV676162 (C06D) showed a pro-oxidative effect against the amastigotes (Fig 2A). However, a pro-oxidative action against the promastigotes was observed upon treatment with Amoxicillin, MMV676600 (C03B), MMV676604 (B03B) and MMV688360 (H03A), while an anti-oxidative effect was shown by Vitamin C, Amphotericin B, MMV688934 (B06A), MMV688958 (G05A), MMV676602 (H02B), MMV652003 (A03B), MMV688797 (E05A), MMV688798 (H05A), MMV202553 (F05A), MMV676057 (E03C), MMV690028 (C05A), MMV688796 (A06A), MMV688514 (E02A), MMV688793 (D05A), MMV676162 (C06D) and MMV688942 (D06A) against the promastigotes (Fig 2B and 2C). However, not all the eighteen selected compounds in this study were assessed for their ROS potential due to insufficient compound. Also, some new compounds were introduced at this stage to be assessed for their potential to generate ROS.

### Externalization of phosphatidylserine (PS) in the outer membranes of *Leishmania donovani* promastigotes treated with the compounds

**Fig 3:**
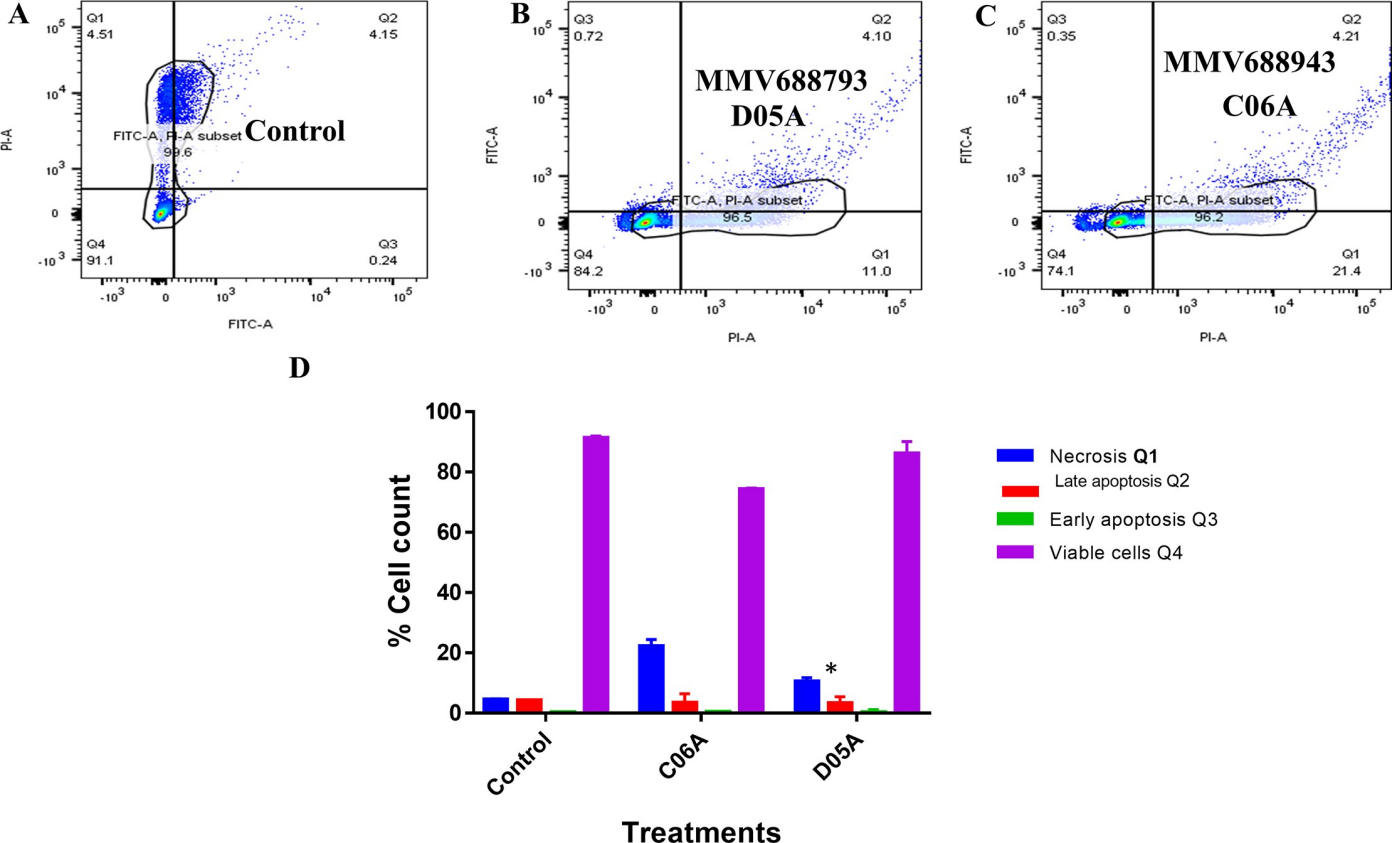
Effect of MMV688793 (D05A) and MMV688943 (C06A) compounds on *Leishmania donovani* promastigotes programmed cell death. (A) Untreated cells (Negative control) and cells treated with (B) MMV688793 (D05A) (2x IC_50_). (C) MMV688943 (C06A) (2x IC_50_). (D) Percentage cell quantification of parasites in the respective stages. Statistical significance was determined using 2-way ANOVA by comparing the treated and the control. Significance was set at P<0.05. These are representative profiles of three independent experiments.

The apoptotic and necrotic potentials of two of the cytocidal compounds [MMV688793 (D05A) and MMV688943 (C06A)] were evaluated against the promastigotes of *Leishmania donovani* to determine their possible cause of death upon treatment with compounds for 24 hrs (Fig 3A–3D). These two compounds were chosen for this assay due to their high therapeutic index aside being cytocidal in action. Here, none of the compounds showed any significant effects (p-value = 0.9926) on the externalization of phosphatidylserine (PS) in the outer membrane of the promastigotes which is an index of their apoptotic potentials (Fig 3B and 3C). However, there was about 6.5% and 17% more necrotic cells for MMV688793 (D05A) and MMV688943 (C06A) respectively compared to that of the negative control (untreated parasites) (Fig 3D).

### Cell cycle analysis of *Leishmania donovani* promastigotes treated with the compounds

**Fig 4:**
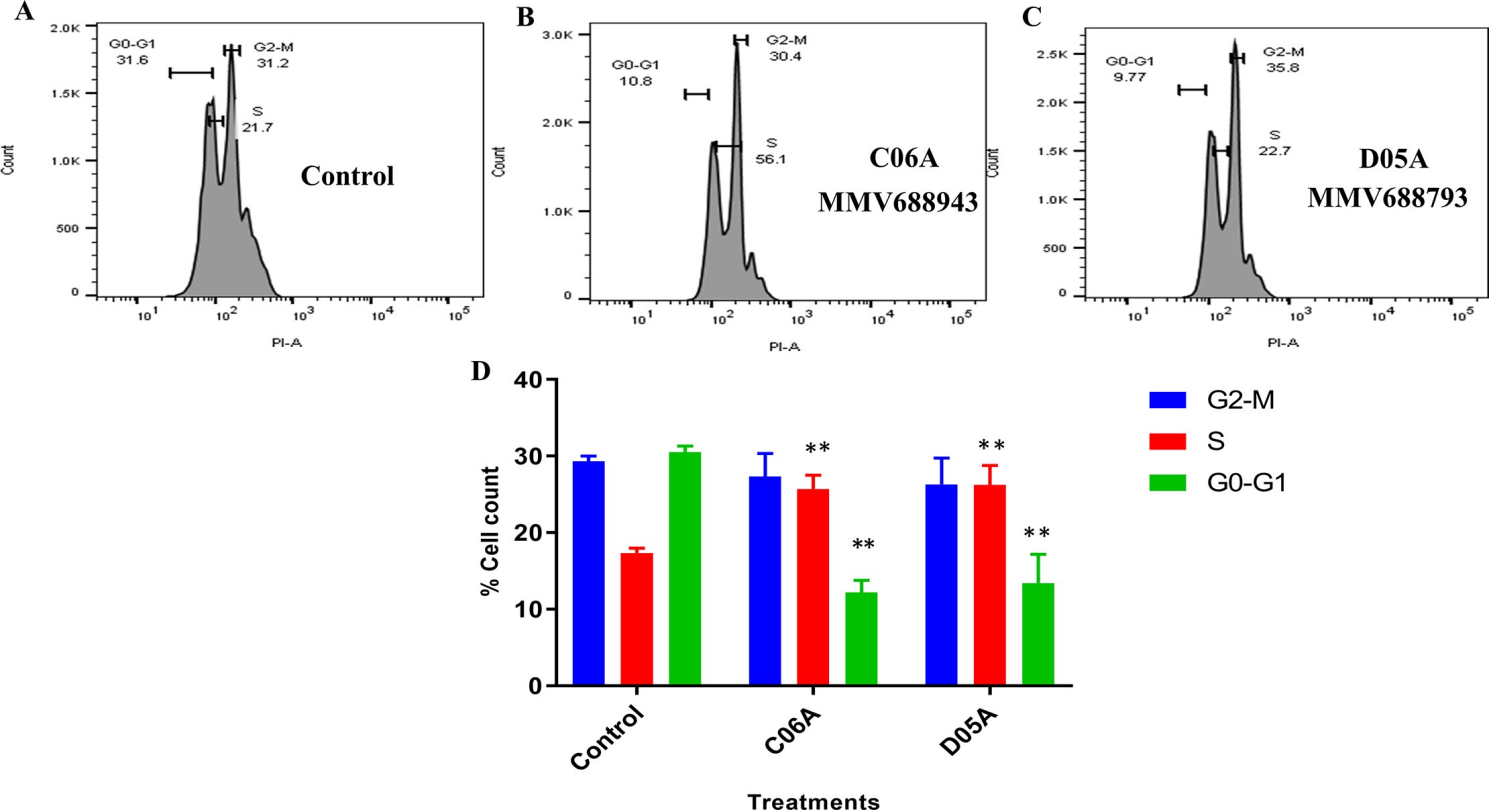
Cell cycle progression of *Leishmania donovani* promastigotes. Flow cytometry histograms of (A) Untreated (B) MMV688943 (C06A) (C) MMV688793 (D05A). Promastigotes were incubated with the compounds at 2x IC_50_ for 24 hrs. Untreated promastigotes were used as the negative control. (D) Cell quantification percentile of each cell cycle phase. The Student’s t test was used to determine statistical significance at *P<0*.*05*. All data shown are representative of three independent experiments.

The two cytocidal compounds with highest therapeutic indices [MMV688793 (D05A) and MMV688943 (C06A)] were assessed for their effect on cell cycle progression of *Leishmania donovani* promastigotes. Upon treatment with the compounds, there were changes in the number of cells at the G0-G1, S and G2-M phases of the cell cycle (Fig 4A–4D). We observed a 1.5- fold increase in parasites at the S phase for both MMV688793 (D05A) and MMV688943 (C06A), and 2.3- and 2.5 -fold decrease in parasites at the G0-G1 for MMV688793 (D05A) and MMV688943 (C06A) respectively. However, there were differences in the number of cells observed at the G2-M phase for both compounds compared with the untreated control. This showed a marked decrease in the ability of most of the cells to enlarge in size (at the G0-G1 phase) and initiate a corresponding division to give rise to as many daughter cells as the untreated. There however, seemed to be an increase in the DNA content at the S-phase, which did not translate into new daughter cells at the G2-M phase. Thus, resulting in a decreased cell population in the treated compared to the untreated parasites (negative control) (Fig 4A–4D).

### Effects of compounds on the morphological and mitochondrial integrity of *Leishmania donovani*

**Fig 5:**
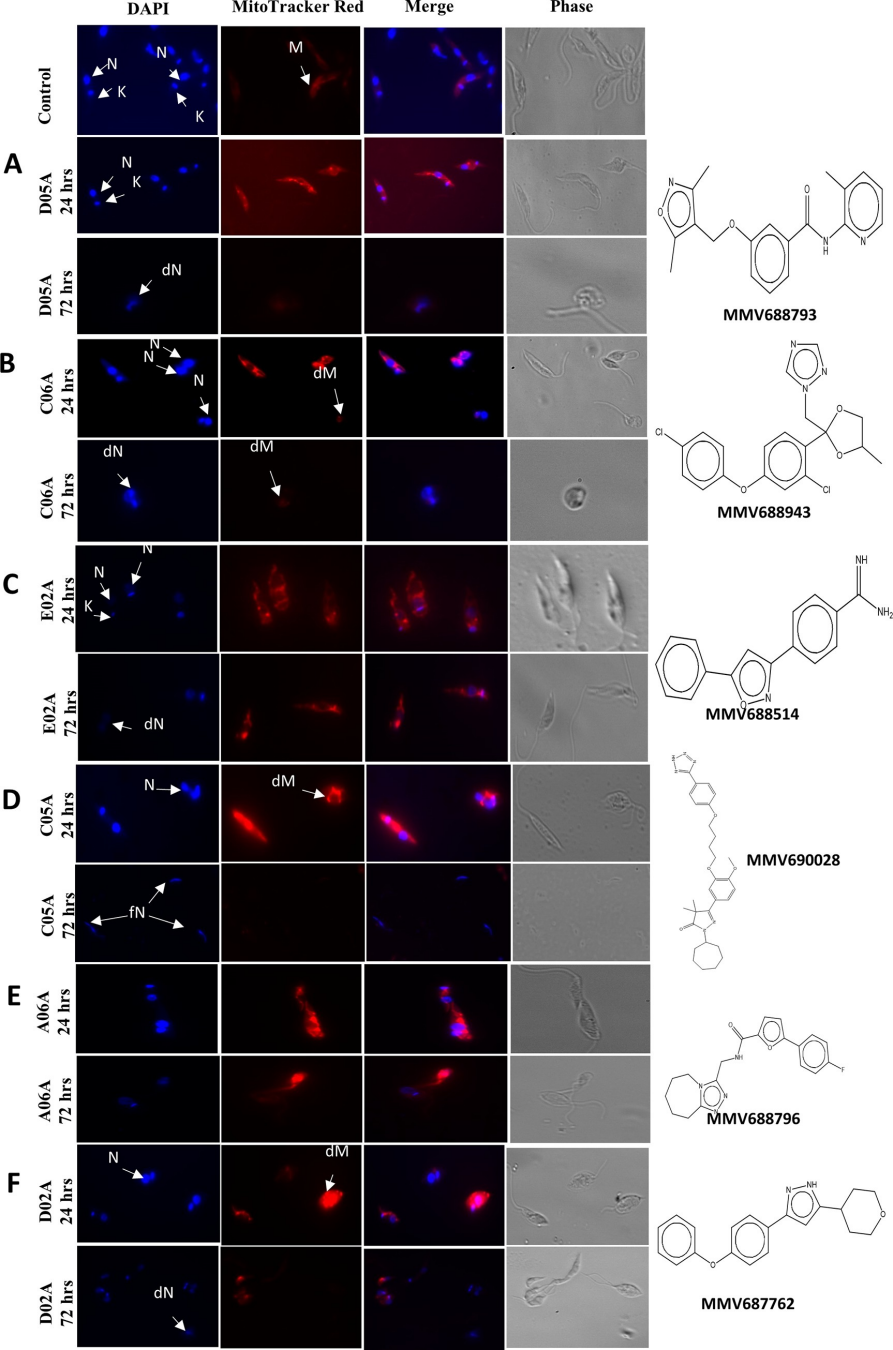
The effects of compounds on the promastigote nuclear / kinetoplast DNA and Mitochondria morphology. **(A)** MMV688793 (D05A) (**B**) MMV688943 (C06A) (**C**) MMV688514 (E02A) (**D**) MMV690028 (C05A) (**E**) MMV688796 (A06A) (**F**) MMV687762 (D02A). Promastigotes were treated with the MMV compound for 24 hrs, 48hrs and 72 hrs. They were stained with DAPI and MitoTracker Red, then examined by phase contrast microscopy and fluorescence microscopy at x1000. Key: M = Normal Mitochondrion; dM = Degenerated Mitochondrion; N = Nucleus; dN = degenerated nucleus; K = Kinetoplast; fN = fragmented Nuclei DNA.

*Leishmania donovani* promastigotes treated with the eighteen MMV compounds showed some abnormalities in their mitochondrial integrity, shape, sizes and nuclear/ kinetoplast DNA morphology (Fig 5A–5F). The figures shown are a representative of the various aberrations observed amongst the eighteen MMV treated cells. Treatment with MMV688793 (D05A) for 24 hrs did not show any obvious abnormalities but upon incubation for 72 hrs, 90% of the nuclear/ kinetoplast DNA were degenerated, the slender shape of the promastigote was completely lost, the mitochondria were degenerated and most of the fields had total parasite clearance (Fig 5A). Treatment with MMV688943 (C06A) at 24 hrs incubation period showed 60% of the cells with lost kinetoplast DNA and the nuclear DNA being enlarged when compared with the negative control (untreated parasites). After 72 hrs incubation, we observed parasite clearance in most fields with a few cells showing complete distortion in shape, degenerated nuclear DNA and mitochondrial degeneration (Fig 5B). Promastigotes treated with MMV688514 (E02A) did not show marked morphological changes after 24 hrs incubation. However, they had their kinetoplast DNA diminished when compared with the untreated parasite control, which degenerate after 72 hrs (Fig 5C). Treatment with MMV690028 (C05A) after 24 hrs incubation showed a complete distortion of cell shape in 50% of the parasites observed with their mitochondria distorted and nuclear and kinetoplast DNA degenerated. Furthermore, after 72 hrs incubation, the cells were completely degenerated with almost no intact cell left for assessment (Fig 5D). Upon treatment of promastigotes with MMV688796 (A06A) compound, after 24 hrs incubation, 50% of the cells showed a slight enlargement in the size of their nuclear and kinetoplast DNA when compared with the untreated parasite control. Then after 72 hrs incubation, most of the few cells left lost their kinetoplast DNA and with their mitochondria degenerated (Fig 5E). Finally, treatment with MMV687762 (D02A) compound showed a degeneration in the mitochondria integrity even after 24 hrs incubation and the degeneration of the nuclear and kinetoplast DNA continued till after 72 hrs incubation (Fig 5F).

## Discussion

In this study, we report the bioactive potencies of some selected pathogen box compounds showing growth inhibition and eventual cell death against *Leishmania donovani* parasites. These parasites were treated with eighteen selected MMV compounds and growth inhibitory concentrations (IC_50_s) ranging from 0.12 –>6.25 μg/ml were recorded against the promastigotes and 0.13- >6.25 μg/ml against the amastigotes stages of the parasite. However, five of the compounds screened (MMV676600 (C03B), MMV676602 (H02B), MMV688514 (E02A), MMV652003 (A03B) and MMV676604 (B03B)) have earlier being reported to have bioactivity against *Leishmania infantum* amastigotes but with no report on their activity against *Leishmania donovani*; with IC_50_s ranging from 0.9–4.1μM while nine others (MMV688796 (A06A), MMV688943 (C06A), MMV687762 (D02A), MMV688793 (D05A), MMV676162 (C06D), MMV676159 (A07E), MMV688797 (E05A), MMV688958 (G05A), MMV099637 (H04A) and MMV688798 (H05A)) were reported to have low potency with IC_50_s ranging from 44.7- >64 μM. Similarly, the antitrypanosomal activity of some of these compounds have been established against *Trypanosoma brucei brucei*, *T*. *cruzi* and *T*. *brucei rhodesiense*; with some of them showing good growth inhibitory potentials [23]. The most active compounds against the promastigotes of *Leishmania donovani* in this study were 12 of the 18 compounds with IC_50_s ranging between 0.12–0.39 μg/ml, while the least active were 6 with IC_50_s ranging between 0.44 - >6.25 μg/ml; with four of the compounds being active against both forms of the parasite. Furthermore, the etiological relationship between *L*. *donovani* and *L*. *infantum* has been reported to be similar, in that, they are both implicated as causative agents of visceral leishmaniasis in the old world but with *L*. *infantum* being further established as one of the species causing oro-mucosal leishmaniasis also known as the mucocutaneous leishmaniasis [24–26].

In a bid to evaluate the cytotoxicity of these compounds, the therapeutic indices of these test compounds were investigated using the murine macrophage cell line and an index range of 16–537 were obtained for eleven of the MMV compounds. This shows good selectivity of these compounds against the *L*. *donovani* parasites *in vitro*, however, the non-selective compounds amongst them had therapeutic indices as poor as 0.03 which is unacceptable for any lead drug candidate. The haemolytic potentials of the compounds was shown to be nil against the human red blood cells even at the highest concentration tested indicating the safety of some of these compounds as possible drug candidates. The compounds tested showed a cytostatic effect on the parasites aside five (MMV688793- D05A, MMV688943- C06A, MMV676604—B03B, MMV676602—H02B and MMV676162—C06D), meaning the parasites were dying while the drug was bioavailable but once there is a drug withdrawal, the parasites resuscitates and growth resumes. However, with the five cytocidal compounds, there were no growth reversal even after drug withdrawal because the parasites were lysed. The importance of identifying drug candidates against leishmaniasis with good selectivity, high solubility property and readily bioavailability at target sites can never be underscored. Recently, a series of water-soluble ferrocenylquinoline derivatives, targeting *Leishmania donovani* were reported; CQFC1 was shown to be the most efficacious of the molecules and it did not induce the resistance-conferring genes in laboratory adapted resistant *L*. *donovani* lines; what is likely in most conventional drugs [27]. In the same vein, the development of a new clinical candidate for visceral leishmaniasis (DNDI-0690) has been reported, which displayed a remarkable 100% oral bioavailability in mice and delivered a 96% parasite burden reduction when dosed at 50 mg/kg in a *Leishmania donovani* mouse model of visceral leishmaniasis [28].

The growth kinetic pattern of the compounds showed that it had time-dependent killing on the parasites. This was observed as the population of the parasites rapidly decreased with time against what was obtainable with the negative control (untreated parasites). In this study, we suggest that the MMV688793- D05A, MMV688943- C06A, MMV676604—B03B, MMV676602—H02B and MMV676162—C06D are better drug candidates than the cytostatic ones because they conferred a lasting therapeutic effect on the parasites than the others (which could still be candidates in combination therapy).

The externalization of phospholipid phosphatidylserine (PS) in the outer membrane of the promastigotes upon treatment with the cytocidal compounds reveals that the cell necrosis recorded on the parasites at the earlier period of incubation was mediated by apoptosis; which has been reported in this group of parasites [29]. The treated parasite cells when compared with the untreated cells showed a very wide margin of cell necrosis attributable to the externalization of PS in the outer membrane of the parasite cells. This was observed upon the conjugation of Annexin V- FITC dye and the binding of Propidium iodide to the apoptotic cells [30]. Here, an apoptosis trigger is a possible mechanism of action of the two cidal compounds against *Leishmania donovani*.

The cell cycle aberrations shown in the mitotic division of the treated promastigotes reveals a marked cell cycle arrest in the G0-G1 and the S phases. Upon incubation of the parasite cells with the cytocidal compounds, the enlargement of organelles at the G0-G1 phase was low when compared with the untreated parasite cells, however, there seemed to be duplication of organelles at the S phase which didn’t translate into new daughter cells. These could be attributed to the profound mitochondrial disruption observed in this study, which is essential for centrosome formation, spindle fibers formation and eventual polarization of single copied organelles resulting in the equatorial invagination of the mother cell and final division into two daughter cells [31]. Therefore, the cell death shown by the action of these compounds can be attributed to the disruptive effect of the compounds on the cell cycle progression of the parasite cells.

The morphological studies of the promastigotes upon treatment with the eighteen MMV compounds revealed the nature of the nuclear and Kinetoplastid DNA (kDNA) after incubation for 24 hrs and 72 hrs to be abnormal. The compounds showed a time dependent effect on the parasites, where 24 hrs incubation in most of the compounds, resulted in the kDNA loss, leaving only the nuclear DNA present. After a 72 hrs incubation, there was complete degeneration of both the nuclear and the kDNA. The mitochondrion was shown to undergo some distortion in shape and eventual degeneration as time progressed in the treatment. The essentiality of the mitochondria in the survival of the *Leishmania* parasite has been demonstrated earlier [32, 33] thus, any aberration in its integrity results in the eventual death of the parasite cells.

Oxidative stress has been implicated as a cause of cell death in many living systems. It is known to arise due to an imbalance between the production and accumulation of some reactive oxygen species (ROS) that are metabolic by-products of certain biological processes. Oxidative stress once increased in a living system, causes damage to cellular entities like proteins, lipids and DNA. It has been reported to be one of the major causes of several diseases like Parkinson’s disease, multiple sclerosis lesions, cutaneous leishmaniasis etc [34–36]. In this study four of the eleven MMV compounds tested for ROS generation potentials against the amastigotes of *Leishmania donovani* showed a pro-oxidative potential comparable to that of amoxicillin and the remaining seven were anti-oxidative. However, of the seventeen MMV compounds tested against the promastigotes of *Leishmania donovani* for their ROS generation potential, only three were pro-oxidative while the remaining fourteen were anti-oxidative.

We have reported here the antileishmanial activity and safety of eighteen selected pathogen box compounds with some of their possible mechanisms of action against the *Leishmania donovani* promastigotes and amastigotes. We suggest the good candidacy of some of them as possible leads in chemotherapy against leishmaniasis. Interestingly, one of the compounds tested is a known broad-spectrum fungicide; Difenoconazole (MMV688943-C06A) with a proven preventive and curative usage against some diseases in fruits, vegetables, cereals and other field crops. It acts by inhibition of demethylation during ergosterol synthesis [37, 38]. In this study we suggest its repurposing as an antileishmanial drug candidate due to its selective bioactivity against *L*. *donovani*.

## Supporting information

S1 Fig Concentration-response curves for the promastigotes of *Leishmania donovani*. Antileishmanial activity of nine of the eighteen MMV compounds against the promastigote stage of the parasite monitored by MTT assay. All data shown are the representation of three independent experiments.
(TIF)

S2 Fig Concentration-response curves for the promastigotes of *Leishmania donovani*. Antileishmanial activity of nine of the eighteen MMV compounds against the promastigote stage of the parasite monitored by MTT assay. All data shown are the representation of three independent experiments.
(TIF)

S3 Fig Concentration-response curves for the amastigotes of *Leishmania donovani*. Antileishmanial activity of nine of the eighteen MMV compounds against the amastigote stage of the parasite monitored by MTT assay. All data shown are the representation of three independent experiments.
(TIF)

S4 Fig Concentration-response curves for the amastigotes of *Leishmania donovani*. Antileishmanial activity of nine of the eighteen MMV compounds against the amastigote stage of the parasite monitored by MTT assay. All data shown are the representation of three independent experiments.
(TIF)

S5 Fig Concentration-response curves for the RAW 264.7 macrophage cell line. Cytotoxicity profile of nine of the eighteen MMV compounds tested against the RAW 264.7 macrophage cell line using the MTT assay. All data shown are the representation of three independent experiments.
(TIF)

S6 Fig Concentration-response curves for the RAW 264.7 macrophage cell line. Cytotoxicity profile of nine of the eighteen MMV compounds tested against the RAW 264.7 macrophage cell line using the MTT assay. All data shown are the representation of three independent experiments.
(TIF)

S7 Fig Concentration-response curves for the haemolytic effect on the red blood cells. Haemolysis profile of nine of the eighteen MMV compounds tested against the human red blood cells. All data shown are the representation of three independent experiments.
(TIF)

S8 Fig Concentration-response curves for the haemolytic effect on the red blood cells. Haemolysis profile of nine of the eighteen MMV compounds tested against the human red blood cells. All data shown are the representation of three independent experiments.
(TIF)
