# Supplementary figures and images for: Some novel antileishmanial compounds inhibit normal cell cycle progression of Leishmania donovani promastigotes and exhibits pro-oxidative potential
Source: PLoS One. 2021 Nov 22;16(11):e0258996. doi: 10.1371/journal.pone.0258996 (PMC8608343; doi:10.1371/journal.pone.0258996)

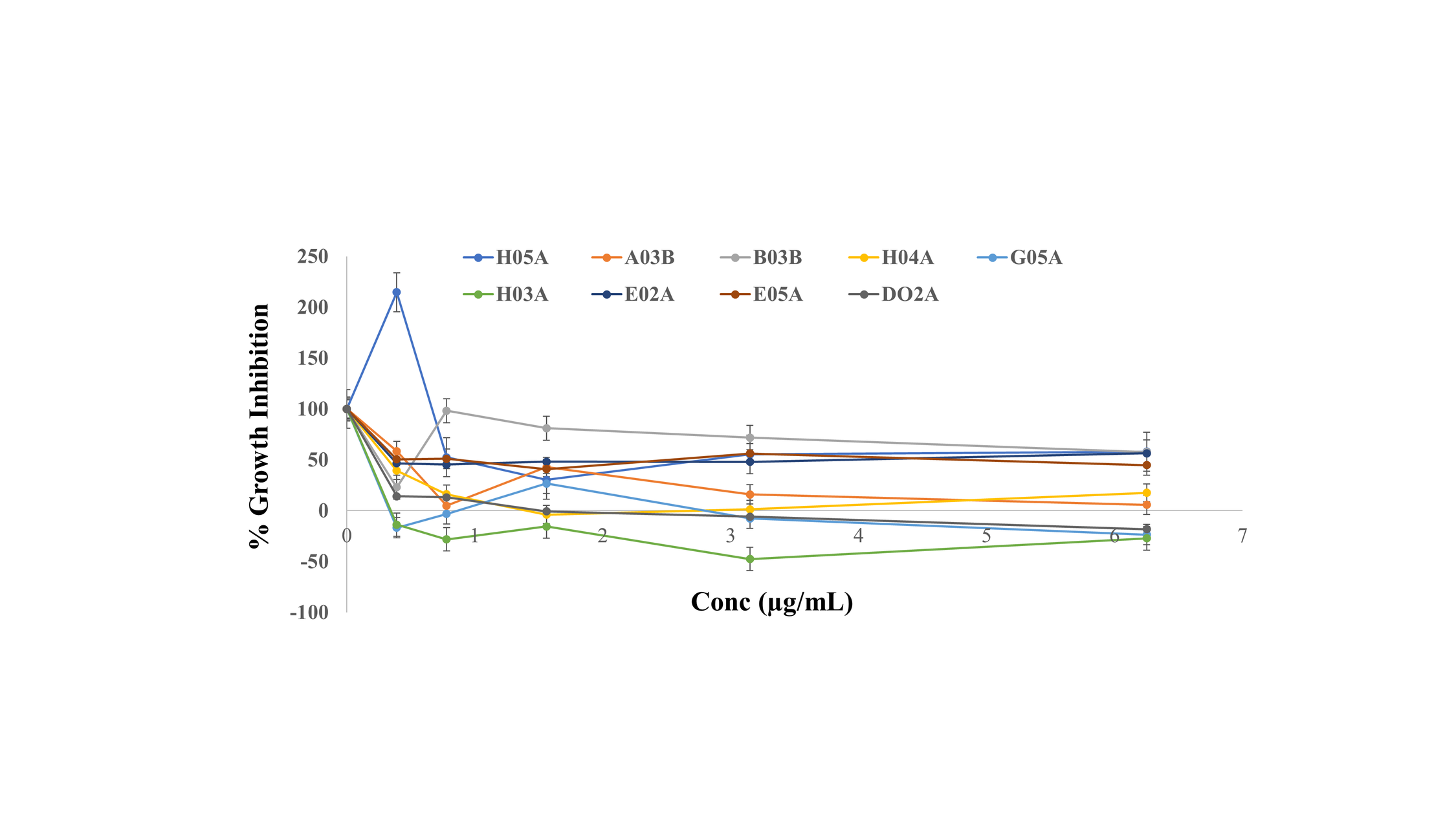

Supplement: S1 Fig — Antileishmanial activity of nine of the eighteen MMV compounds against the promastigote stage of the parasite monitored by MTT assay. All data shown are the representation of three independent experiments. (TIF) [file pone.0258996.s001.tif]

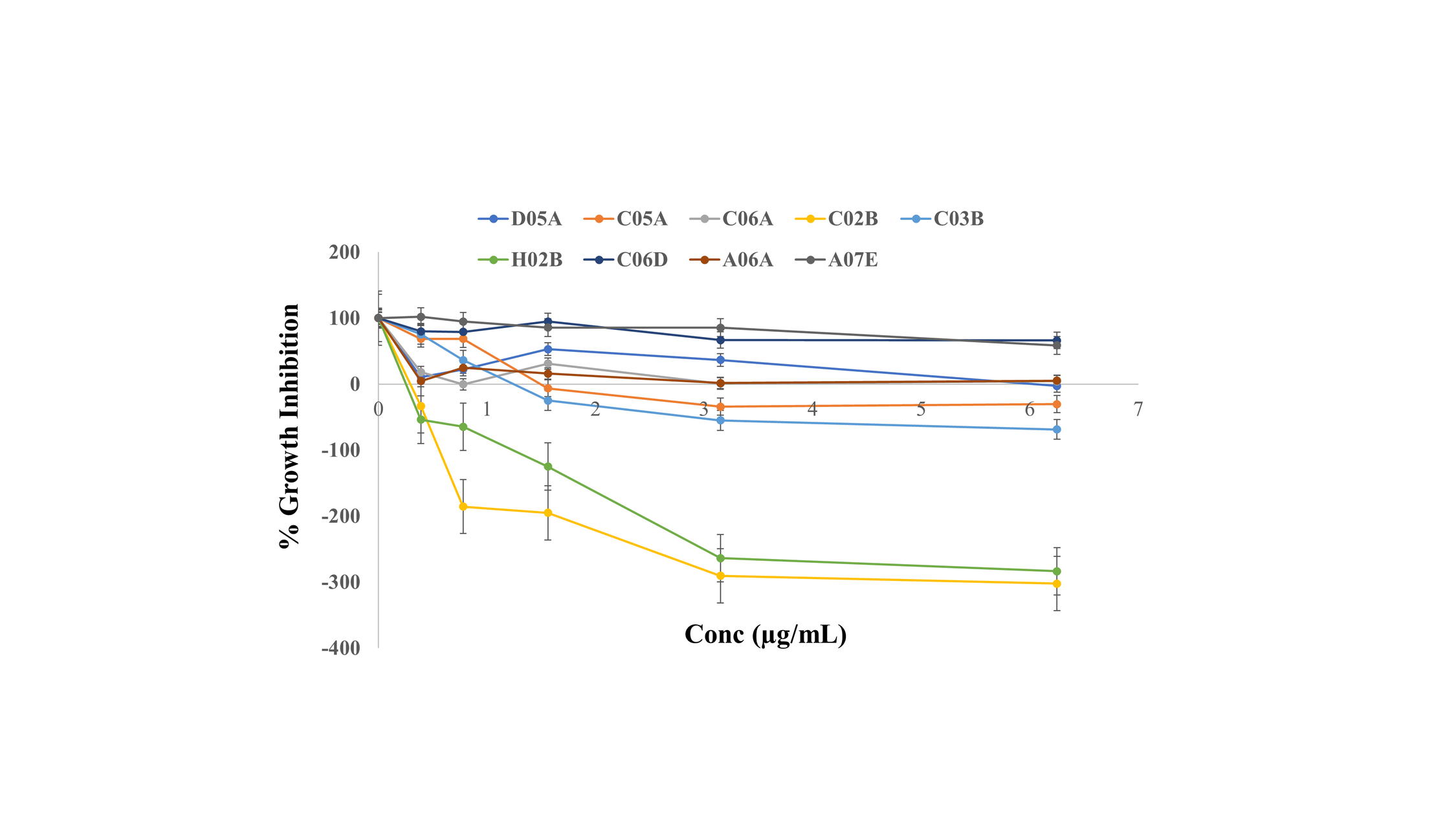

Supplement: S2 Fig — Antileishmanial activity of nine of the eighteen MMV compounds against the promastigote stage of the parasite monitored by MTT assay. All data shown are the representation of three independent experiments. (TIF) [file pone.0258996.s002.tif]

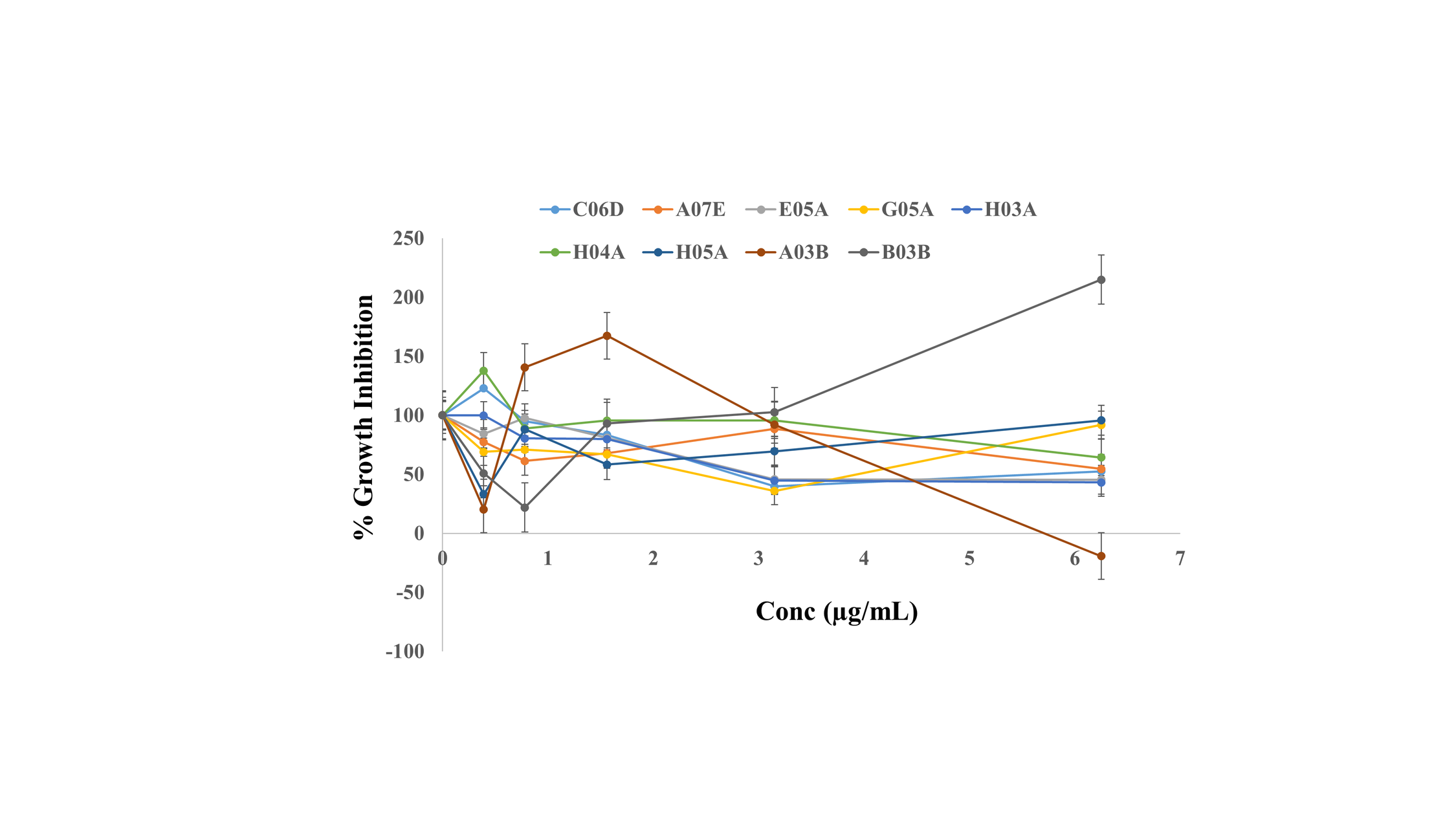

Supplement: S3 Fig — Antileishmanial activity of nine of the eighteen MMV compounds against the amastigote stage of the parasite monitored by MTT assay. All data shown are the representation of three independent experiments. (TIF) [file pone.0258996.s003.tif]

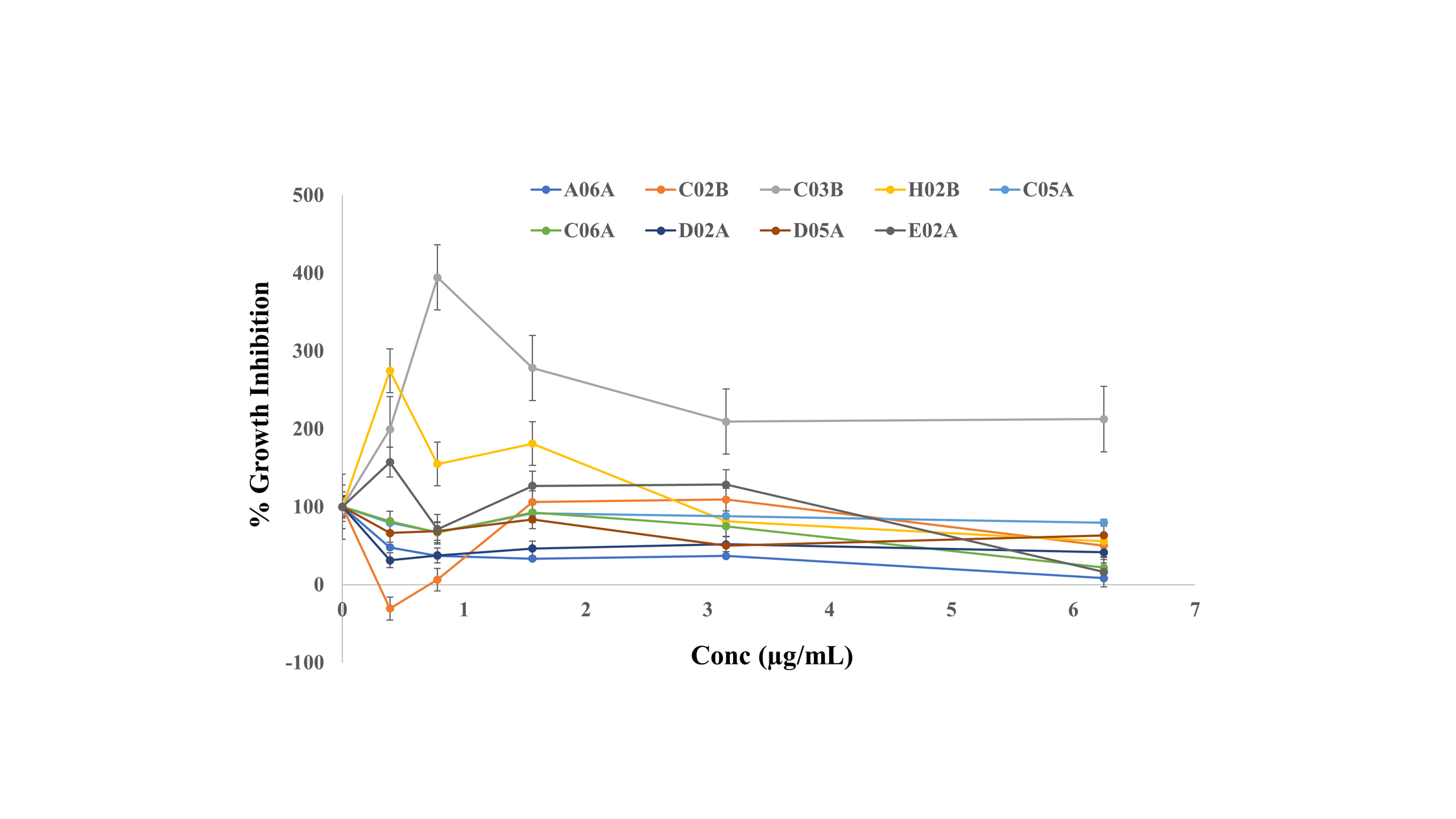

Supplement: S4 Fig — Antileishmanial activity of nine of the eighteen MMV compounds against the amastigote stage of the parasite monitored by MTT assay. All data shown are the representation of three independent experiments. (TIF) [file pone.0258996.s004.tif]

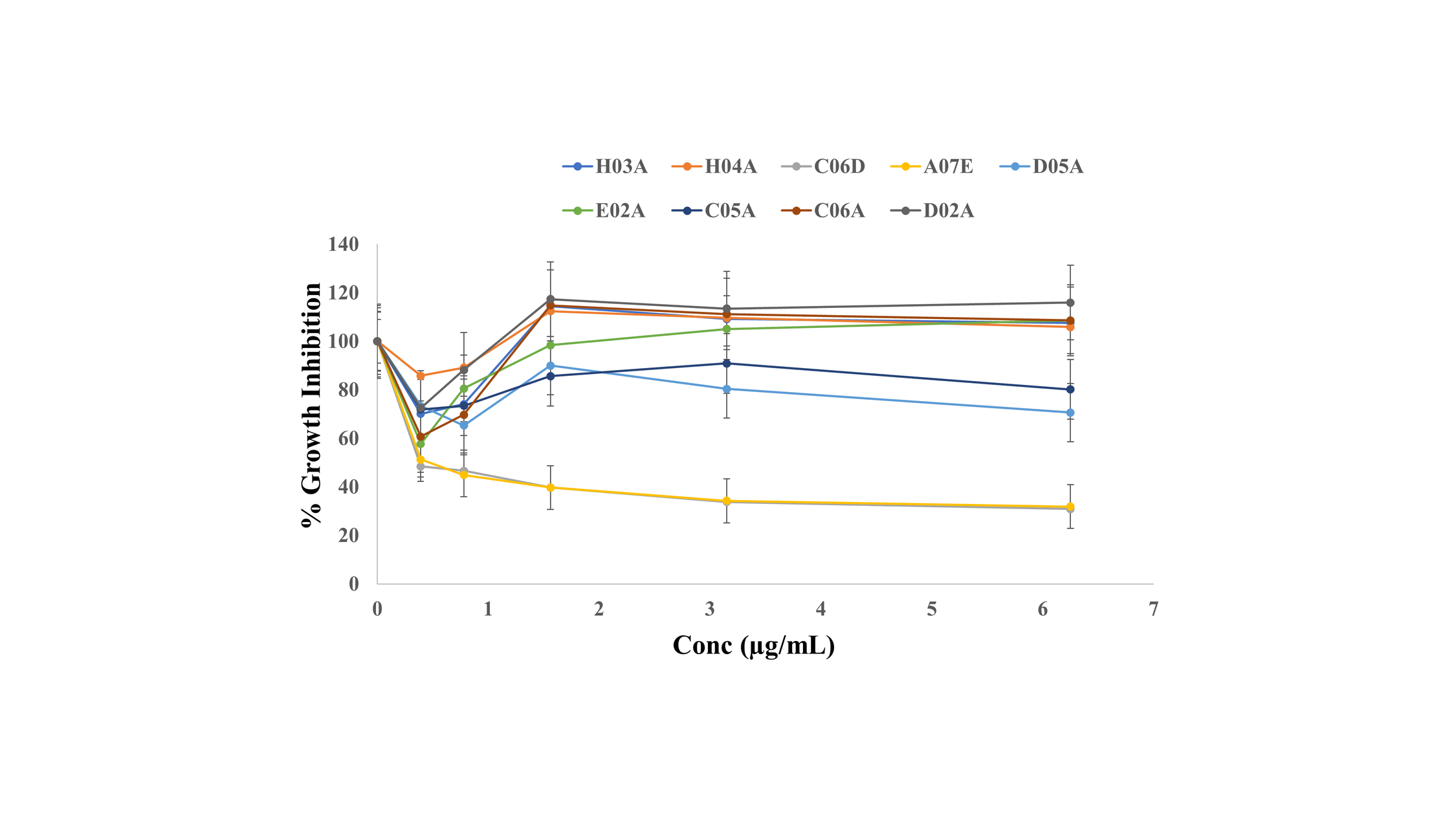

Supplement: S5 Fig — Cytotoxicity profile of nine of the eighteen MMV compounds tested against the RAW 264.7 macrophage cell line using the MTT assay. All data shown are the representation of three independent experiments. (TIF) [file pone.0258996.s005.tif]

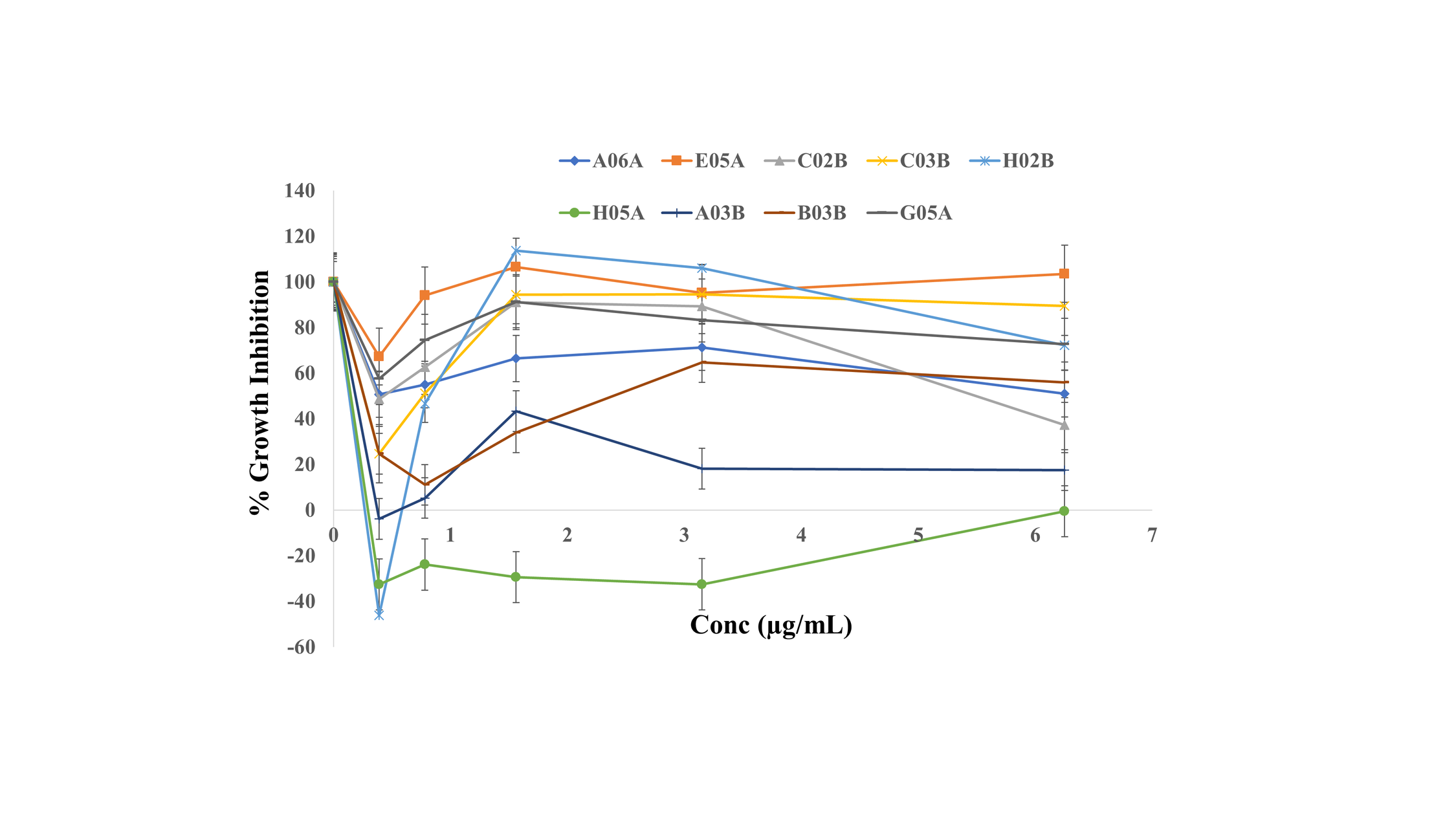

Supplement: S6 Fig — Cytotoxicity profile of nine of the eighteen MMV compounds tested against the RAW 264.7 macrophage cell line using the MTT assay. All data shown are the representation of three independent experiments. (TIF) [file pone.0258996.s006.tif]

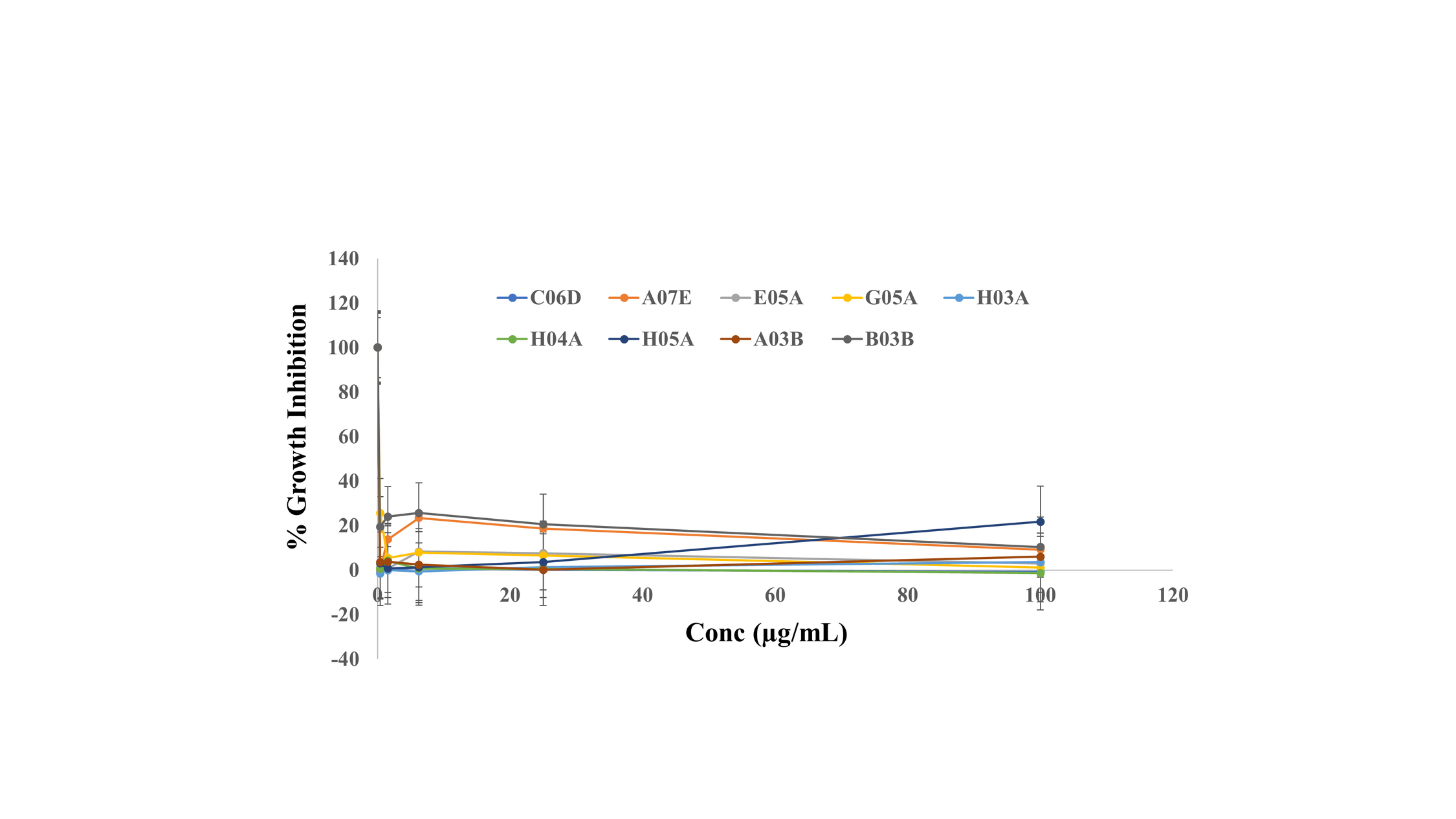

Supplement: S7 Fig — Haemolysis profile of nine of the eighteen MMV compounds tested against the human red blood cells. All data shown are the representation of three independent experiments. (TIF) [file pone.0258996.s007.tif]

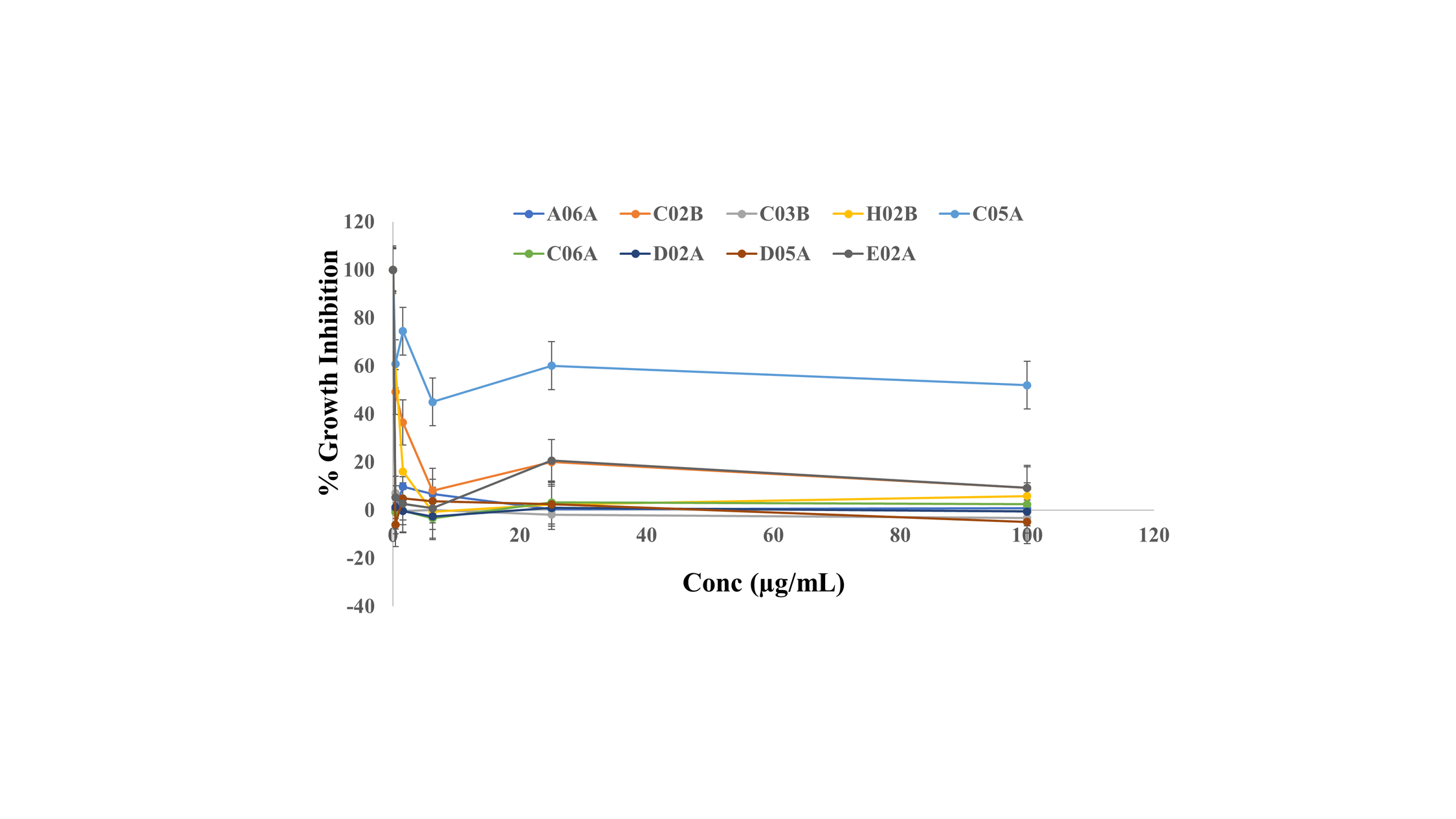

Supplement: S8 Fig — Haemolysis profile of nine of the eighteen MMV compounds tested against the human red blood cells. All data shown are the representation of three independent experiments. (TIF) [file pone.0258996.s008.tif]
